# Generation of a murine SWATH-MS spectral library to quantify more than 11,000 proteins

**DOI:** 10.1038/s41597-020-0449-z

**Published:** 2020-03-26

**Authors:** Chuan-Qi Zhong, Jianfeng Wu, Xingfeng Qiu, Xi Chen, Changchuan Xie, Jiahuai Han

**Affiliations:** 1 State Key Laboratory of Cellular Stress Biology, Innovation Center for Cellular Signaling Network, School of Life Sciences, Xiamen University, Xiamen, China; 2 Department of Gastrointestinal Surgery, Zhongshan Hospital of Xiamen University, Xiamen, China; 3 Medical Research Institute, Wuhan University, Wuhan, China; 4 SpecAlly Life Technology Co., Ltd, Wuhan, China

**Keywords:** Bioinformatics, Proteomics, Proteomic analysis, Mouse

## Abstract

Targeted SWATH-MS data analysis is critically dependent on the spectral library. Comprehensive spectral libraries of human or several other organisms have been published, but the extensive spectral library for mouse, a widely used model organism is not available. Here, we present a large murine spectral library covering more than 11,000 proteins and 240,000 proteotypic peptides, which included proteins derived from 9 murine tissue samples and one murine L929 cell line. This resource supports the quantification of 67% of all murine proteins annotated by UniProtKB/Swiss-Prot. Furthermore, we applied the spectral library to SWATH-MS data from murine tissue samples. Data are available via SWATHAtlas (PASS01441).

## Abstract (summary)

**Table:** 

Measurement(s)	Mouse Protein • mass spectrum • spectral library
Technology Type(s)	mass spectrometry • combined ms-ms + spectral library search
Sample Characteristic - Organism	Mus musculus

Machine-accessible metadata file describing the reported data: 10.6084/m9.figshare.11968230

## Background & Summary

Data-independent acquisition (DIA) mass spectrometry is an emerging approach for consistent and accurate protein quantification across multiple samples. Sequential Windowed Acquisition of All Theoretical Fragment Ion Mass Spectra (SWATH-MS) is one of the DIA methods that has been employed to produce highly reproducible and complete quantitative results^1–3^. This property of SWATH-MS enables the general application of SWATH-based quantitative proteomics in biological research and clinical biomarker studies^4–6^.

SWATH-MS data analysis can be accomplished by two strategies, spectral library-based targeted analysis approach and library-free analysis method. A spectral library is usually generated through data-dependent acquisition (DDA) measurement of the peptides which are recorded by SWATH-MS. Library-free methods such as DIA-Umpire^7^, Group-DIA^8^, PECAN^9^, and MSPLIT-DIA^10^, though not requiring a spectral library, have been reported to be less sensitive than spectral library-based approach^10–12^. The depth and composition of the spectral library typically determine the outputs of SWATH-MS. Although a sample-specific spectral library can be generated, large previously established spectral libraries can offer more identifications and reduce the amount of samples and MS measurement time. The comprehensive spectral libraries for organisms such as human^13^, drosophila^14^, and zebrafish^15^ have been published.

Because of its close genetic and physiological similarities to humans, the mouse has been the premier mammalian model system for genetic and biomedical research. Additionally, murine cell lines are extensively utilized in molecular mechanism research^16^. Considering the widespread use of mice in these research, a comprehensive mouse SWATH-MS spectral library would be beneficial to the studies by quantitatively comparing the protein contents across multiple murine samples.

The mouse genome encodes about 22,480 protein-coding genes, among which 17,094 mouse protein-coding genes have human orthologues. Although a murine spectral library has been generated in a published study^17^, the proteome coverage of the spectral library is relatively low (6,652 of 20,002 in PANTHER database) and the detail of the spectral library regarding the numbers of proteotypic peptides and the number of peptides per protein are unclear. What’s more, the published library does not contain DDA files from murine cell lines. Although cell lines were originally derived from a given tissue, the gene expression profiles change during the establishment of the cell line and during cell culture *in vitro*. The inclusion of the DDA data from the murine cell line in the murine spectral library should increase proteome coverage.

**Fig. 1:**
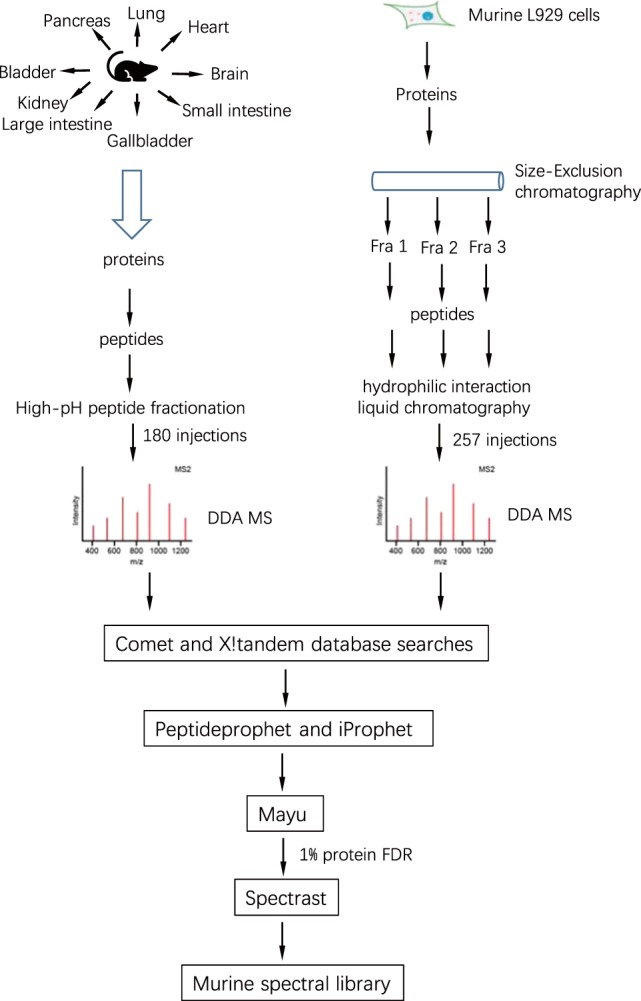
Sample preparation and data analysis workflows used in the generation of the spectral library. L929 cell lysates were first fractionated with size-exclusion chromatography and digested with trypsin. The resulting peptides were fractionated with HILIC (Hydrophilic Interaction Liquid Chromatography). The tissue samples were digested with trypsin and the peptides were fractionated with high-pH chromatography. The peptide fractions were dissolved in 0.1% formic acid containing iRT peptides, which were analyzed using shotgun MS. The DDA files were searched with X!Tandem and Comet, and results were combined with iProphet. The combined results were filtered with 1% protein FDR and made a consensus spectral library with Spectrast software.

Here we present a large-scale murine spectral library to support protein quantification by SWATH-MS. It was generated by combining the 437 DDA runs from peptide samples derived from the murine L929 cell line and 9 murine tissues (257 runs for L929 and 180 runs for tissues). The murine L929 cell line DDA data were collected through protein fractionation followed by extensive peptide fractionation^8^, while tissue DDA data were acquired using high-pH peptide fractionation (Fig. 1 and Table 1). The murine spectral library consists of 243,043 proteotypic peptides which correspond to 11,340 proteins. We further show that the murine spectral library can be applied in tissue SWATH-MS data analysis and provide more identifications than the internal library which was built directly from SWATH-MS data.

**Table 1:** DDA runs in each datasets.

Sample	Protein fractionation	Peptide fractionation	MS samples
L929 cell line	Size exclusion chromatography	HILIC	257
Pancreas	NA	High-pH RP-HPLC	20
Lung	NA	High-pH RP-HPLC	20
Heart	NA	High-pH RP-HPLC	20
Brain	NA	High-pH RP-HPLC	20
Small intestine	NA	High-pH RP-HPLC	20
Gallbladder	NA	High-pH RP-HPLC	20
Large intestine	NA	High-pH RP-HPLC	20
Kidney	NA	High-pH RP-HPLC	20
Bladder	NA	High-pH RP-HPLC	20

## Methods

### Mouse tissue sample preparation

Three C57BL/6 mice of postnatal 50 days were used for tissue dissection. All animal experimental protocols were approved by the Institutional Animal Care and Use Committee at Xiamen University. Tissues were snap-frozen in liquid nitrogen upon dissection. Tissues were homogenized in 4% SDC/10 mM TCEP/40 mM CAA/100 mM Tris-HCl pH 8.5 on the Scientz-48 High Throughput Tissuelyser (Scientzbio, Ningbo, China). Protein supernatants were collected by centrifugation, and protein concentrations were assayed with Pierce 660 nm protein assay reagent (Thermo). Proteins were heated at 60 °C for 30 min to denature the proteins and carbamidomethylate thiols. 4% sodium deoxycholate (SDC) was diluted to 1% SDC, and trypsin (Sigma) was added into reactions at the ratio of 1:50. The digestions were performed at 37 °C overnight. Subsequently, 1% trifluoroacetic acid (TFA) was added and SDC precipitations were removed by centrifugation. The peptides were desalted using in-house made SDB-RPS StageTips^18^. The StageTips were washed with 100 μl 1%TFA/ isopropanol (ISO) and subsequent 100 μl 0.2%TFA/H_2_O. The peptides were eluted with 80% acetonitrile/5% NH_3_.H_2_O. The buffers were evaporated using Speedvac at 45 °C.

### Sample preparation for the murine L929 cell line

The detailed method for murine cell line L929 sample preparation has been described^8^. Briefly, L929 cells were lysed with 2% SDS 100 mM Tris-HCl pH 8.5, and proteins were fractionated using size exclusion chromatography. 0.1 ml of the cell lysate containing 10 mg of total protein was loaded onto a Superdex 200 10/300 GL column (GE Healthcare Bio-Sciences AB, Uppsala) equilibrated with TNS buffer composed of 0.1 M Tris-HCl, pH 8.0 buffer, 0.1 M NaCl and 0.2% SDS. Proteins were eluted with TNS buffer and fractions were collected according to elution profile. Total 8 fractions were collected.

The resulting protein fractions were digested using FASP protocol^19^. The tryptic peptides were fractionated with HILIC (hydrophilic interaction liquid chromatography) column. HILIC was performed using a 1260 HPLC system (Agilent) with a TSKgel Amide-80 HILIC column (2.0 × 150 mm, 5 μm; Tosoh Biosciences, Tokyo, Japan) at a flow rate of 150 μl/min. Two buffers were used for the gradient: buffer A, 90% ACN containing 0.005% TFA, and buffer B, 0.005% TFA. Peptides were resuspended in 200 μl of 70% ACN and then injected into the HILIC Amide-80 column via a 200 μl loop with a flow rate of 150 μl/min. The gradient used is as follows: 0% buffer B at time 0 min, 11% buffer B at 5 min, 29% buffer B at 20 min, 95% buffer B at 45 min, hold 95% buffer for 5 min, and finally 0% buffer B at 55 min. Fractions were collected according to elution profile and dried.

### High-pH fractionation of peptides

High-pH fractionation was performed on an Agilent Infinity 1260 system. About 200 μg peptides were injected for each organ. The peptides were separated at 25 °C on a TechMate C18 reversed-phase column with a diameter of 0.5 mm, length of 150 mm particle, size of 3 μm, and pore size of 12 nm. A 60 min gradient was delivered as followed: 5–25% Buffer B (Buffer B: 10 mM ammonium formate, 40% acetonitrile, 12.5% ammonia solution; Buffer A: 20 mM ammonium formate, pH 10) in 20 min, then increased to 45% in 40 min and to 90% in 1 min. The resulting 60 fractions were pooled to 20 fractions. The pooling procedure was performed as followed: fraction x was pooled with fractions x + 10 and x + 20. The pooled fractions were desalted with SDB-RPS StageTips and evaporated using vacuum centrifugation.

### Data-Dependent acquisition of peptide samples

Peptides were dissolved in 0.1% formic acid containing iRT peptides (Hangzhou Go Top Peptide Biotech Co., Ltd., China). MS analysis was performed on a TripleTOF 5600 (Sciex) mass spectrometer coupled to NanoLC Ultra 2D Plus (Eksigent) HPLC system. Peptides were first bound to a 300SB-C18 trap column (ZORBAX, Agilent). The analytical column was a 35 cm × 75 μm in-house pulled emitter-integrated column packed with Magic C18 AQ 3-μm 200- Å resin. The peptide separation was performed using a linear 60 min gradient from 2–35% buffer B (buffer A 0.1% (V/V) formic acid, 5% DMSO in H_2_O, buffer B 0.1% (V/V) formic acid, 5% DMSO in acetonitrile). In one cycle, one MS1 scan was followed by 20 MS2 scans. MS1 scan collected 350–1250 m/z for 250 ms and MS2 scan collected 100–1,800 m/z for 50 ms. Exclusion time for precursor ions selection is 20 s. Ions were fragmented for MS2 experiment in the collision cell using a collision energy according to the equation of a doubly charged peptide, ramped ± 15 V from the calculated collision energy.

### SWATH-MS analysis of tissue samples

The peptides derived from tissue samples were dissolved in 0.1% FA containing iRT peptides. The setting of nano liquid chromatography was the same as described in DDA analysis except for 180-min gradient. Mass spectrometer was operated in SWATH mode, and MS1 scan records a 350–1250 m/z range for 250 ms and a 100–1800 m/z range was recorded for 33.3 ms in the high-sensitivity mode MS2 scan. One MS1 scan was followed by 100 MS2 scans, which covered a precursor m/z range from 400–1200. The variable windows of SWATH-MS were “399.5–409.9, 408.9–418.9, 417.9–427.4, 426.4–436, 435–443.6, 442.6–450.8, 449.8–458, 457–464.8, 463.8–471.1, 470.1–476.9, 475.9–482.8, 481.8–488.6, 487.6–494, 493–499, 498–504.4, 503.4–509.3, 508.3–514.3, 513.3–519.2, 518.2–524.2, 523.2–529.1, 528.1–534.1, 533.1–539, 538–543.5, 542.5–548.5, 547.5–553, 552–558, 557–562.5, 561.5–567, 566–571.5, 570.5–576, 575–580.5, 579.5–585, 584–589.5, 588.5–594, 593–598, 597–602.5, 601.5–607, 606–611.1, 610.1–615.6, 614.6–620.1, 619.1–624.6, 623.6–628.6, 627.6–633.1, 632.1–637.6, 636.6–642.1, 641.1–646.6, 645.6–651.1, 650.1–655.6, 654.6–660.1, 659.1–665.1, 664.1–669.6, 668.6–674.5, 673.5–679, 678–684, 683–688.5, 687.5–693.4, 692.4–698.4, 697.4–703.3, 702.3–708.7, 707.7–713.7, 712.7–719.1, 718.1–724.5, 723.5–729.9, 728.9–735.3, 734.3–740.7, 739.7–746.5, 745.5–751.9, 750.9–757.8, 756.8–763.6, 762.6–769.5, 768.5–775.3, 774.3–781.2, 780.2–787, 786–793.3, 792.3–800.1, 799.1–806.4, 805.4–813.1, 812.1–820.3, 819.3–827.5, 826.5–835.2, 834.2–843.3, 842.3–851.4, 850.4–859.9, 858.9–868.9, 867.9–878.4, 877.4–888.3, 887.3–899.1, 898.1–910.3, 909.3–922.9, 921.9–936, 935–949.5, 948.5–963.4, 962.4–978.7, 977.7–994.9, 993.9–1015.6, 1014.6–1042.2, 1041.2–1070.1, 1069.1–1100.7, 1099.7–1140.7, 1139.7–1196.5”.

### Bioinformatics analysis

#### Building the murine spectral library

The DDA raw files (wiff) were converted to centroided mzML files using qtofpeakpicker^20^ tool in Proteowizard software (V.3.0.447)^21^. The mzML files were searched with X!Tandem^22^ (Version 2013.06.15.1, native and k-score^23^) and Comet^24^ (Version 2017.01) which has been integrated into TPP (Trans-Proteomic Pipeline, Version 5.0)^25^ against an UniprotKB/Swiss-Prot murine protein database (downloaded at 20190627) which contains 34,279 entries including reversed sequence decoys, contaminant proteins (contaminant protein sequences are obtained from maxquant software) and iRT peptide sequences. The search engines parameters were set as followed. The parent and product ions mass tolerance is 50 ppm and 0.1 Da respectively. Carbamidomethyl (C) was set as a fixed modification and oxidation (M) as a variable modification. The pep.xml search results were validated and scored using PeptideProphet^26^ with parameters –OARPd -dDECOY and combined by iProphet^27^ with parameters DECOY = DECOY. Mayu (version 1.07)^28^ was used for protein FDR control. The iProphet probability 0.996973 was selected, which corresponded to protein FDR 0.009765. The peptide ions passing the 1% protein FDR were input into SpectraST^29^ for library building with CID-QTOF setting. The retention time of peptides in sptxt file was replaced with iRT time using spectrast2spectrast_irt.py script (https://github.com/msproteomicstools/msproteomicstools), and the peptides used for retention time normalization were endogenous peptides (CiRT^30^) or spiked-in iRT peptides^31^. The sptxt file was made consensus non-redundant spectral library with the iRT retention time using spectraST.

#### Building the internal spectral library

SWATH-MS files were converted to centroided mzXML files using qtofpeakpicker tool as described above. Centroided mzXML files were analyzed with DIA-Umpire. DIA-Umpire was run with default setting except for BoostComplementaryIon = false. mgf files were converted to mzML files using msconvert (Proteowizard V.3.0.447). mzML files were subjected to database searches and spectral library generation as described in DDA files above.

#### Targeted analysis of SWATH-MS data using OpenSWATH-PyProphet-TRIC workflow

The workflow was performed as previously described^4^. SWATH-MS files were converted to 32-bit profile mzXML files using msconvert (Proteowizard V.3.0.447). The consensus sptxt files were converted to tsv using spectrast2tsv.py script (available at https://github.com/msproteomicstools/msproteomicstools) which then converted to TraML file with TargetedFileConverter tool which was integrated into OpenMS software (Version 2.2.0)^32^. In OpenSWATH analysis, ciRT peptide^30^ and iRT peptides^31^ were used for retention time normalization. The XIC extraction window was 20 min. An extended version of PyProphet^33,34^ (PyProphet-cli v0.19) was employed for FDR estimation. For each tissue dataset, 1% protein FDR at the global level was applied. The filtered results were input into TRIC software for cross-run alignment. The parameters in TRIC^35^ were set as followed: –method LocalMST –realign_method lowess_cython –max_rt_diff 60 –mst:useRTCorrection True –mst:Stdev_multiplier 3.0 –target_fdr 0.01–max_fdr_quality 0.05.

#### Protein quantification

Protein quantification was conducted as previously described^4^. The TRIC results were used for protein inference and quantification. Peptide intensities were obtained directly from TRIC output results. All identified peptides from the specific protein were ranked by the average intensity in all runs. Subsequently, the top three intense peptides of the specific protein were selected and the sum of these three peptide intensities represented the protein intensity in each run. Where <3 peptides were detected, the available peak groups were summed.

## Data Records

The raw mass spectrometry DDA files for library generation and SWATH-MS files, the search results (pepXML), the consensus spectral library are deposited on the PeptideAtlas with identifier PASS01441 and can be accessed at http://www.peptideatlas.org/PASS/PASS01441 ^36^.

## Technical Validation

### False discovery rate control at protein level

**Fig. 2:**
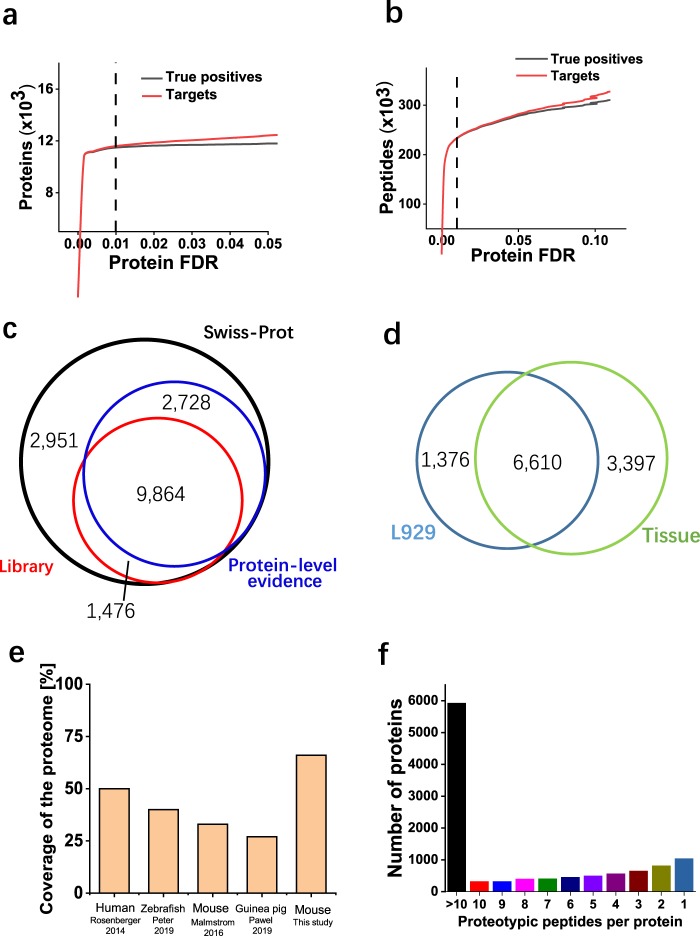
Characteristics of the murine spectral library. (**a**) True positive (black) and all protein identifications (red) as a function of protein FDR. The vertical dashed line was protein FDR of 0.01 determined by MAYU software. (**b**) True positive (black) and all peptide identifications (red) as a function of protein FDR. The vertical dashed line was protein FDR of 0.01 determined by MAYU software. (**c**) Overlap of murine proteins in UniProtKB/Swiss-Prot, a subset annotated with protein-level evidence and the murine spectral library. (**d**) Coverage of the proteome for SWATH-MS spectral libraries of different species. The numbers of proteome coverage were directly taken from the cited publication^13,15,17,38^. (**e**) The number of proteotypic peptides per protein in the murine spectral library.

False discovery rate (FDR) is the metric for global confidence assessment of a large-scale proteomics dataset. For the purpose of spectral library generation, the dataset composed of a large number of runs should be strictly filtered. We used MAYU software to filter the dataset at 1% protein FDR. It is difficult to know the true positive hits for a mass spectrometry dataset. To estimate the expected number of true positive and false positive protein identifications, MAYU employs a hypergeometric model that takes the number of target and decoy protein identifications and the total number of protein entries in the dataset as input. As shown in Fig. 2a, true positive proteins have reached saturation at 1% protein FDR. On the contrary, the true positive peptides kept increasing at this cutoff (Fig. 2b). This suggested that the higher number of false positive protein identification would be accepted if 1% peptide FDR were applied, which is consistent with published results^37^. We applied 1% protein FDR to the whole dataset to retain only high-quality protein identifications.

### Properties of the murine spectral library

To demonstrate the proteome coverage of the murine spectral library, we compared the proteins included in the murine spectral library with those in UniProtKB/Swiss-Prot (version 2019_08) and those with evidence on protein-level. 78.3% (9,864 of 12,592) of proteins with protein-level evidence were included in our library, and 1,476 additional proteins with protein-level evidence were provided by the murine library (Fig. 2c). Among these 1,476 proteins, 27.2% (401 of 1,476) of them have one distinct peptide. These single-hit peptides have high-quality MS2 spectra (Supplementary Fig. 1). 41.1% (607 of 1,476) of them are identified by 2–5 unique peptides, while 17.2% (254 of 1,476) of them contain 6–10 peptides. 15.9% (234 of 1,476) of proteins are identified by more than 10 unique peptides. About 12% (1,376 of 11,383) proteins were exclusively provided by L929 DDA files, while 29.8% (3,397 of 11,383) provided by tissue DDA files (Fig. 2d). In comparison with the UniProtKB/Swiss-Prot, the murine library contains 66.6% (11,340 of 17,019) of all proteins, which is the largest proteome coverage among all published spectral libraries^13,15,17,38^ (Fig. 2e). Table 2 provides an overview of the contents of the murine spectral library. Compared to the human spectral library^13^, almost two times of proteotypic peptides were included in the murine spectral library. To show the coverage of a single protein, we calculated the number of proteotypic peptides per protein. About 52% of the proteins in the library contain >10 proteotypic peptides per protein, and 91% of them contain at least two proteotypic peptides per protein (Fig. 2f).

**Table 2:** Contents in the murine spectral library.

	Proteotypic	Proteotypic and shared
Proteins	11,340	15,408
Peptides	2,43,043	2,57,137
Precursors	2,71,396	2,87,114
Transitions	16,28,376	17,22,684

### Applicability of the murine spectral library for SWATH-MS analysis

**Fig. 3:**
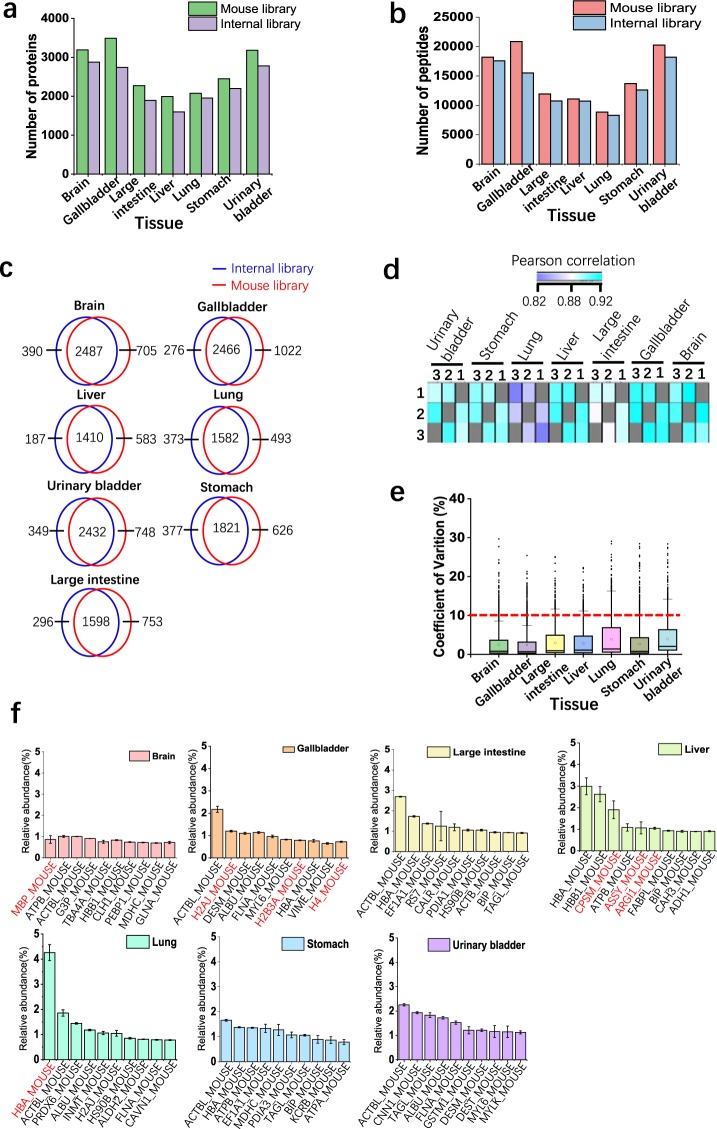
Analyzing tissue SWATH-MS data using the murine spectral library. (**a**) The numbers of quantified proteins at 1% global protein FDR in three technical replicates in seven tissue datasets. The mouse library and the internal libraries were used to analyze SWATH-MS data. (**b**) The numbers of quantified peptides at 1% global protein FDR in three technical replicates in seven tissue datasets. (**c**) Pearson correlation of protein intensities identified in two samples. (**d**) CV of log2-transformed intensities of quantified proteins in three replicates using L929 library. (**e**) The proteins with the top ten highest abundances in each tissue. The protein intensity was normalized with the sum of all protein intensities, and top ten proteins were shown. The tissue-function related proteins were labelled in red (protein entry names are from UniProt/SwissProt database).

To show the usage of the murine spectral library in analyzing SWATH-MS data, we acquired seven mouse tissue samples (brain, gallbladder, large intestine, liver, lung, stomach, urinary bladder) in technical triplicate using SWATH-MS. With the murine library, OpenSWATH was employed for targeted analysis of SWATH-MS data. PyProphet was utilized to control protein FDR, and TRIC was used to retrieve the missing value in the quantitative results. We analyzed SWATH-MS data from seven mouse tissue samples separately, and 1% global protein FDR was applied in all analyses. To evaluate the performance of the murine library, we also used DIA-Umpire to analyze these SWATH-MS data. The mgf files from DIA-Umpire were used to build the internal libraries, which were subjected to OpenSWATH-PyProphet-TRIC workflow analysis. In total, about 2000–3000 proteins and 10,000–20,000 peptides were quantified in each tissue dataset using the murine library (Fig. 3a,b). We examined the overlapping proteins by the two libraries. Generally, at least 75% of proteins identified by two libraries overlapped (Fig. 3c). Although the number of raw files used for generation of the murine library (437 runs) is significantly higher than that of internal libraries (3 runs), the minor increases of peptide and protein identifications by the murine library compared to internal libraries are observed. The limited performance of the comprehensive probably resulted from the relatively low sequence coverage of proteins in the library^13^, which will be improved in the future version.

To evaluate the quality of the murine library-based analysis, we quantitatively compared protein abundance across the different runs in each dataset. Pearson’s correlation coefficients were 0.82–0.92 between any two different runs (Fig. 3d). The correlation for replicates of lung is lower than that for other tissues. This phenomenon is probably attributed to the interferences from the tissue (Supplementary Fig. 2). To further determine quantitative reproducibility, we computed the coefficient of variation (CV) in each tissue dataset. For all tissue dataset, the median CVs of log2-transformed protein abundance were below 10% (Fig. 3e). Collectively, the murine library-based targeted analysis of SWATH-MS exhibited excellent reproducibility in the entire experiment.

With the quantitative protein intensities in each tissue, we examine the relationship between the abundance of proteins and functions of a specific tissue. The normalized abundances of top ten proteins in each tissue were shown in Fig. 3f. Actlb2 (ACTBL_MOUSE) is the most abundant protein among almost all tissues. In brain, MBP (MBP_MOUSE) is the most abundant protein, which is a major component of myelin membrane in the central nervous system^39^. Alpha-globin (HBA_MOUSE) were involved in oxygen transport from the lung to the various peripheral tissues. Consistently, Alpha-globin is the most abundant protein in lung. Three histone proteins (H2AJ_MOUSE, H2B3A_MOUSE and H4_MOUSE) showed up in the top ten proteins of gallbladder, while nearly no histone protein was detected in the top ten proteins in other tissues. Liver is an organ where excess ammonia is removed through the urea cycle in the mitochondria of cells. Accordingly, three enzymes (CPSM_MOUSE, ASSY_MOUSE and ARGI1_MOUSE) that play the key roles in urea cycle occurred in the top ten proteins of liver. These results demonstrate that the murine spectral library can be used for a comprehensive exploration of SWATH-MS data derived from murine samples.

## Usage Notes

### Generating alternative SWATH spectral libraries from the full spectral library

In this study, we generated a 100-VW SWATH-MS assay library from the murine spectral library. However, the murine SWATH-MS assay library with any other window configuration can be easily be performed based on the murine full spectral library using the spectrast2tsv.py script.

### Control of false-discovery rate (FDR)

It is crucial for controlling FDR when analyzing large-scale of SWATH-MS data using a comprehensive spectral library^34^. Therefore, the appropriate workflow including FDR controlling at protein level should be employed when analyzing SWATH-MS data using the spectral libraries especially for very large ones. OpenSWATH-PyProphet-TRIC workflow and the commercial Spectronaut software^40^ meet this requirement.

### Limitations of the murine spectral library

The current spectral library presented here is constructed from 9 murine tissues and one cell line, and the proteins that specifically expressed in other murine tissues may not be included in the murine spectral library. Another concern is about the portability of the spectral library to other platforms such as Orbitrap and Timspro TOF. In this study, DDA runs were collected on the TripleTOF 5600 instrument, which is primarily used for the purpose of analyzing SWATH-MS data. The human spectral library built with DDA runs on TripletOF 5600 has been used for targeted analysis of DIA data acquired on Orbitrap platform^41–43^. However, the analysis results based on the TripleTOF-generated library may be sub-optimal due to the differential fragmentation patterns from distinct MS platforms. The murine spectral library can be applied to DIA data acquired on different platforms, but careful examination of analysis results is required.

## Supplementary information

Supplementary Figure 1.

Supplementary Figure 2.

## Data Availability

The software in this study has been described^34,35,44^. The workflows to analyze SWATH-MS data have been published^45^ and are described on http://www.openswath.org.
