# Supplementary material for: Generation of a murine SWATH-MS spectral library to quantify more than 11,000 proteins
Source: Sci Data. 2020 Mar 26;7:104. doi: 10.1038/s41597-020-0449-z (PMC7099061; doi:10.1038/s41597-020-0449-z)
Supplement: Supplementary file 1 — Supplementary Figure 1. [file 41597_2020_449_MOESM1_ESM.pdf]

Supplementary Figure 1

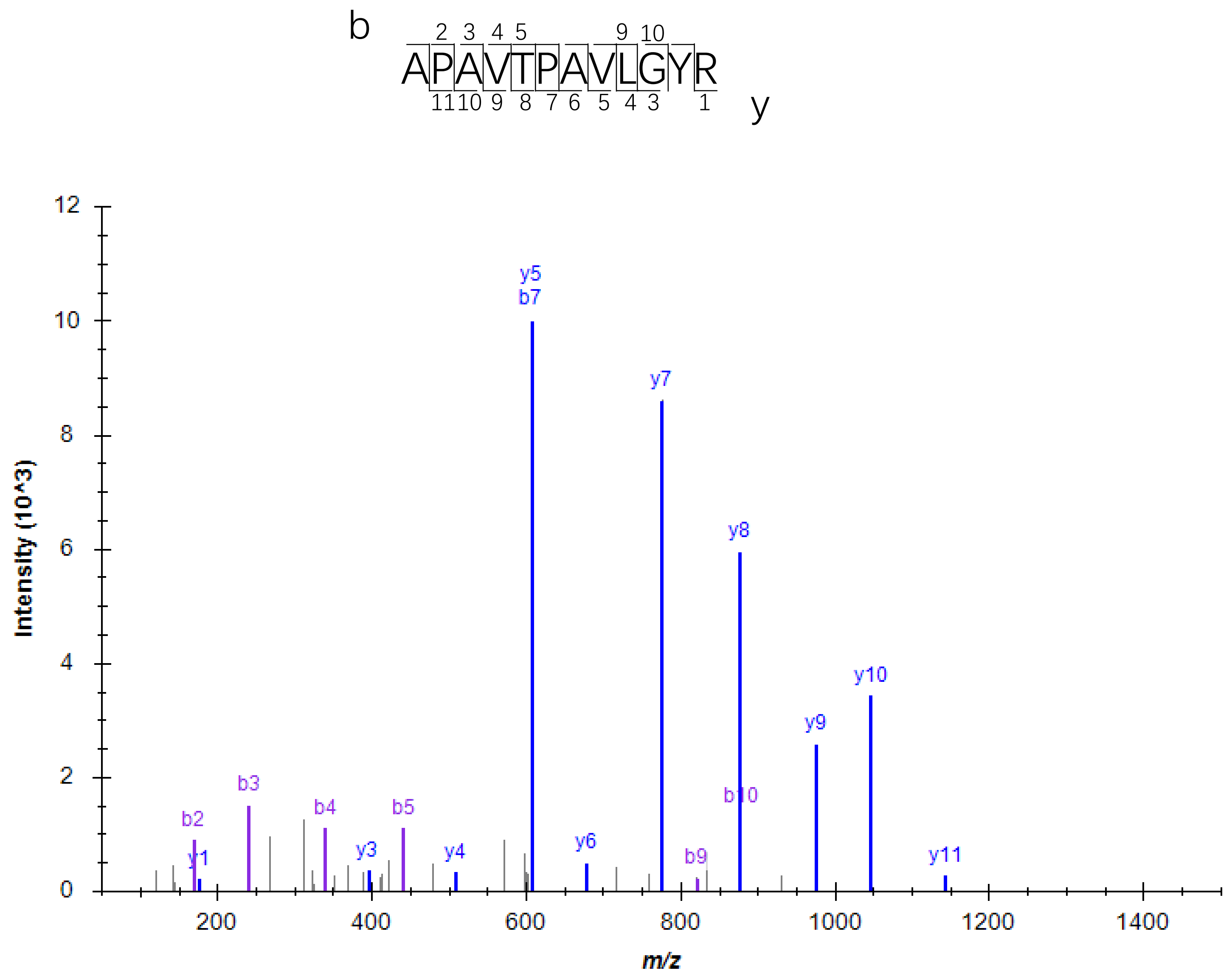

Supplementary Figure 1. The MS2 spectrum for one-hit peptide “APAVTPAVLGYR”. The detected b and y product ions have been labelled.
