# Supplementary material for: Generation of a murine SWATH-MS spectral library to quantify more than 11,000 proteins
Source: Sci Data. 2020 Mar 26;7:104. doi: 10.1038/s41597-020-0449-z (PMC7099061; doi:10.1038/s41597-020-0449-z)
Supplement: Supplementary file 2 — Supplementary Figure 2. [file 41597_2020_449_MOESM2_ESM.pdf]

Supplementary Figure 2

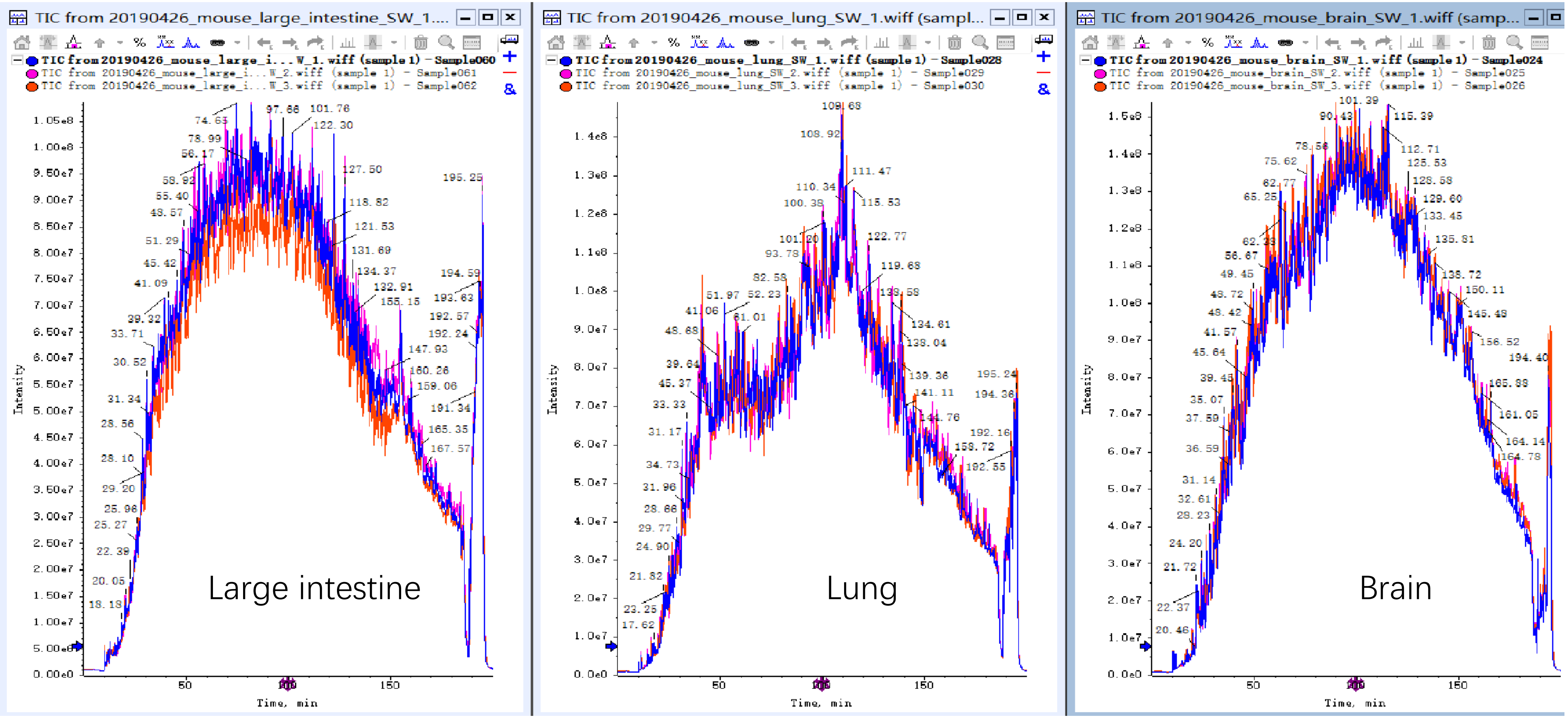

Supplementary Figure 2. The overlay of total ion chromatography (TIC) for three SWATH-MS runs in large intestine, lung and brain dataset.
